# Functional consequences of somatic polyploidy in development

**DOI:** 10.1242/dev.202392

**Published:** 2024-02-28

**Authors:** Gabriella S. Darmasaputra, Lotte M. van Rijnberk, Matilde Galli

**Affiliations:** Hubrecht Institute, Royal Netherlands Academy of Arts and Sciences and University Medical Center Utrecht, Uppsalalaan 8, 3584 CT, Utrecht, the Netherlands

**Keywords:** Cancer, Cell size, Gene expression, Polyploidy

## Abstract

Polyploid cells contain multiple genome copies and arise in many animal tissues as a regulated part of development. However, polyploid cells can also arise due to cell division failure, DNA damage or tissue damage. Although polyploidization is crucial for the integrity and function of many tissues, the cellular and tissue-wide consequences of polyploidy can be very diverse. Nonetheless, many polyploid cell types and tissues share a remarkable similarity in function, providing important information about the possible contribution of polyploidy to cell and tissue function. Here, we review studies on polyploid cells in development, underlining parallel functions between different polyploid cell types, as well as differences between developmentally-programmed and stress-induced polyploidy.

## Teaser

**Summary:** This Review explores the functions of polyploidy during multicellular development, displaying common features of polyploid cell types and highlighting differences between developmentally programmed and induced polyploidy.

## Introduction

The term polyploid refers to cells that contain more than two sets of chromosomes. Within multicellular species, polyploidy can be found at both the organismal level, when germ cells undergo a whole-genome duplication (WGD) and give rise to complete polyploid organisms, or at the sub-organismal level, when only a subset of somatic cells within an otherwise diploid organism become polyploid. The consequences of polyploidization can differ significantly between these different types of polyploidy. Here, we focus on the consequences of polyploidy at the sub-organismal level, outlining the functions of somatic polyploid cells during normal physiology and in disease.

**Fig. 1.**
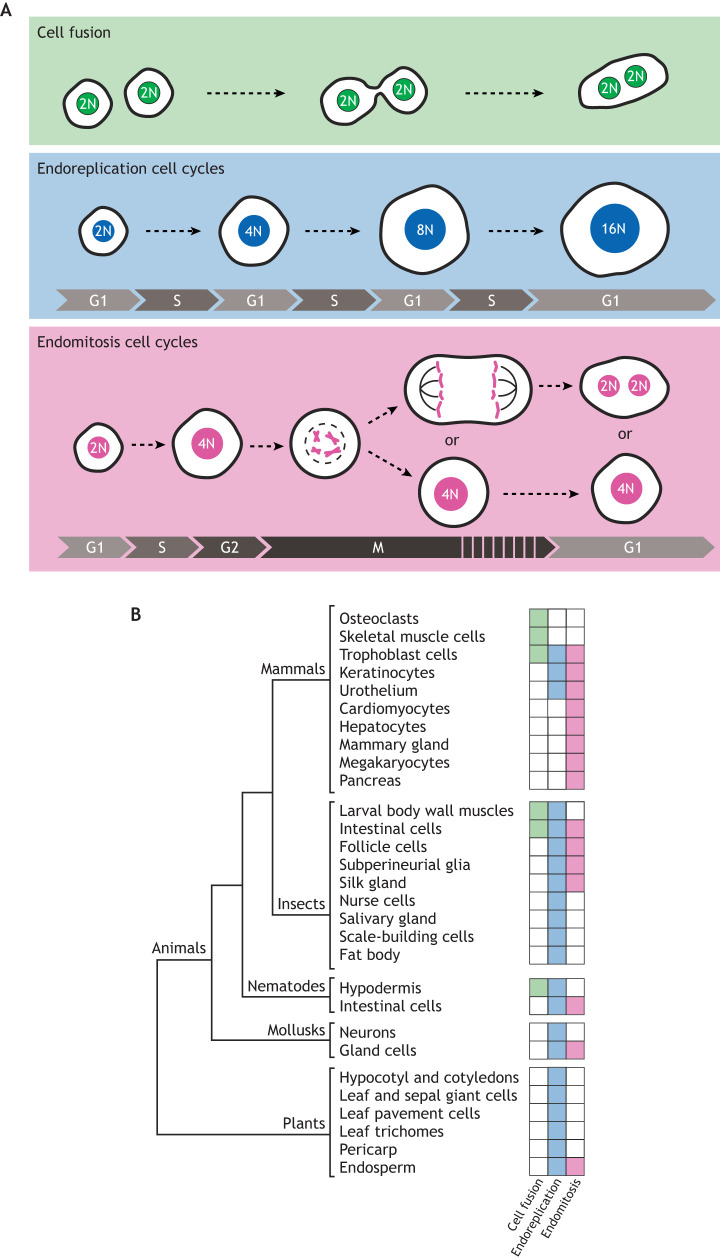
**Overview of polyploid cell formation and occurrence in multicellular organisms.** (A) Polyploid cells can arise by three mechanisms during development: by cell fusion, where two or more diploid cells fuse to form a multinucleated syncytium; by endoreplication, a non-canonical cell cycle in which cells alternate between DNA replication (S phase) and gap (G) phases; by endomitosis, non-canonical cell cycles in which cells enter M phase but do not undergo cellular division. Depending on the timing of M-phase exit, endomitosis can result in either mononucleated or binucleated cells. (B) Overview of polyploid cell types in multicellular species, and whether they arise by cell fusion (green boxes), endoreduplication (blue boxes) or endomitosis (pink boxes).

The first observations of polyploid cells within an otherwise diploid organism were made in the late nineteenth century, shortly after the discovery of chromosomes (Wilson, 1925). Over the past century, studies in plants and insects have greatly contributed to our understanding of how somatic polyploidy arises, which has been extensively reviewed elsewhere (Almeida et al., 2022; Edgar et al., 2014; Hua and Orr-Weaver, 2017). In summary, somatic cells can become polyploid either by cell fusion or by undergoing non-canonical cell cycles in which they replicate their DNA but do not divide into two daughter cells. Many terms have been used to describe these non-canonical cell cycles but, in essence, they can be divided into two types: non-canonical cell cycles in which cells alternate between S and G phase, which we refer to as ‘endoreplication cycles’, and non-canonical cell cycles in which cells undergo all phases of the canonical cell cycle but exit M phase before the initiation or completion of cytokinesis, which we refer to as ‘endomitosis cycles’ (Edgar et al., 2014; Fig. 1A). In some endoreplication cycles, cells do not replicate the whole genome during S phase, which can give rise to polytene chromosomes, in which sister chromatids remain closely associated (Stormo and Fox, 2017). There are also many variations of endomitosis cycles, which depend on the timing of when cells exit M phase. For example, cells can exit M phase either before or after anaphase onset, and thus give rise to either mononucleated or binucleated polyploid cells (Fig. 1A).

The widespread occurrence of polyploid cells within multicellular organisms suggests that somatic polyploidization has evolved independently in many plant and animal species (Fig. 1B). Furthermore, certain cell types use multiple mechanisms to become polyploid; for example, trophoblast cells can either fuse, undergo endomitosis or undergo endoreplication (Ilgren, 1983), *Caenorhabditis elegans* intestinal cells undergo both endoreplication and endomitosis, *Drosophila* muscle cells undergo both cell fusion and endoreplication, and *Drosophila* nurse cells undergo multiple types of endoreplication, giving rise to enormous nuclei containing both polytene and non-polytene organizations during different moments in development (Dej and Spradling, 1999; Demontis and Perrimon, 2009; Hedgecock and White, 1985; Windner et al., 2019), suggesting that being polyploid confers specific advantages for a cell, irrespective of how it is achieved.

In this Review, we discuss key aspects of polyploid cell function in development. We describe common characteristics of naturally occurring polyploid cells, such as their increased cell size and their propensity to generate large amounts of proteins for their environment, as well as their ability to fine-tune gene expression. Finally, we briefly highlight the cellular consequences of stress-induced polyploidy, where cells become polyploid after ectopic stresses such as tissue injury.

## Polyploidy is associated with increased cell and organ sizes

One property of polyploid cells that has long been appreciated is their increased cellular size. Already in the early 1900s, Theodor Boveri manipulated cellular ploidy levels in sea urchins and demonstrated that cells containing more DNA were proportionally larger than cells with less DNA (Boveri, 1905). Since then, the positive correlation between DNA content and cell size has been made across multiple plant and animal species. For example, in the moth *Ephestia kuehniella*, the volume of Malpighian tubule cells and silk gland cells increases more than a thousand-fold as they become highly polyploid during larval development (Buntrock et al., 2012). Similarly, cell size scales with DNA content in mammalian hepatocytes, cardiomyocytes, megakaryocytes and neurons (Epstein, 1967; Katzberg et al., 1977; Penington and Olsen, 1970; Sigl-Glöckner and Brecht, 2017). In plants, positive correlations between ploidy and cell size have been reported in leaf and stem epidermal tissues (Melaragno et al., 1993), as well as in the tomato fruit pericarp (Cheniclet et al., 2005). Fruit cells undergoing the highest number of endoreplication cycles are some of the largest cells found in plants (with diameters ranging between 600 and 1000 µm), suggesting that polyploidy is required to reach very large cell sizes (Bourdon et al., 2010).

Despite the correlation between polyploidization and increases in cell and organ size, a direct causative relationship has only been shown for a limited number of polyploid tissues: in *C. elegans*, polyploidization of the hypodermis is required for animals to reach their correct body size (Lozano et al., 2006); in tomato plants, reducing the ploidy of fruits by downregulation of CCS52A, the plant ortholog of the APC/C activator CDH1, results in smaller size fruits (Bourdon et al., 2010); in *Drosophila melanogaster*, the size of the subperineurial glial cells (SPGs), salivary glands and hair bristles all depend on polyploidization because blocking DNA replication in these tissues decreases cell and organ sizes (Sallé et al., 2012; Szuplewski et al., 2009; Unhavaithaya and Orr-Weaver, 2012; Weng et al., 2003). Conversely, increasing ploidy levels gives rise to larger SPGs and brain lobes in *Drosophila* (Li et al., 2017; Unhavaithaya and Orr-Weaver, 2012), and larger body sizes in *C. elegans* (Lozano et al., 2006). Together, these findings point to a key role of polyploidy in promoting cell and organ growth.

### Mechanistic insights into polyploidy and cellular scaling

The mechanism by which increased cellular ploidies result in larger cells is not well understood. Intuitively, one would argue that increased DNA copy number increases messenger RNA (mRNA) expression, thus leading to more protein production and larger cells. Most studies on gene expression differences between naturally occurring polyploid cells of different ploidies have focused on relative differences in the expression of a subset of genes rather than absolute changes in total mRNA or protein levels between cells of different ploidies (Lu et al., 2007; Richter et al., 2021; Sun et al., 2021; Windmueller et al., 2020). Nonetheless, studies in polyploid plant cell types suggest a linear scaling of genome content with mRNA abundance and cell size (Pirrello et al., 2018; Robinson et al., 2018). Interestingly, increasing cell size has been shown to increase mRNA transcription in yeast, plant and animal cells, thus an increase in mRNA in polyploid cells could also be explained by increased cell sizes (Ietswaart et al., 2017; Padovan-Merhar et al., 2015; Zhurinsky et al., 2010). Moreover, analyses of RNA transcription rates during G2 phase of the cell cycle, when the genome is also doubled, have shown limited dependency on DNA dosage (Padovan-Merhar et al., 2015; Skinner et al., 2016; Voichek et al., 2016; Yunger et al., 2010). Transcriptional buffering mechanisms compensate for gene dosage imbalances after DNA replication, ensuring that transcription of ubiquitously expressed genes does not fluctuate across the cell cycle (Huang et al., 2021; Voichek et al., 2016, 2018). Thus, doubling the amount of cellular DNA does not necessarily result in twice as much mRNA production, which makes it unclear how polyploidy stimulates cell growth. One explanation could be that, in polypoid cells, such buffering mechanisms are not present. Alternatively, cellular growth is not directly a consequence of increased mRNA production but rather the consequence of increased protein biosynthesis. In support of this, ribosomal RNA (rRNA) synthesis increases drastically during polyploidization in tomato plants and *C. elegans* intestines (Bourdon et al., 2012; Uppaluri et al., 2016). Taken together, additional investigation of the scaling between DNA content, RNA transcription, protein translation and cell growth, together with experimental manipulations of ploidy and cell sizes in diverse polyploid cell types, will be needed to gain a deeper understanding of how polyploidy controls cell growth.

### Polyploidy allows organs to grow while maintaining tissue barriers

**Fig. 2.**
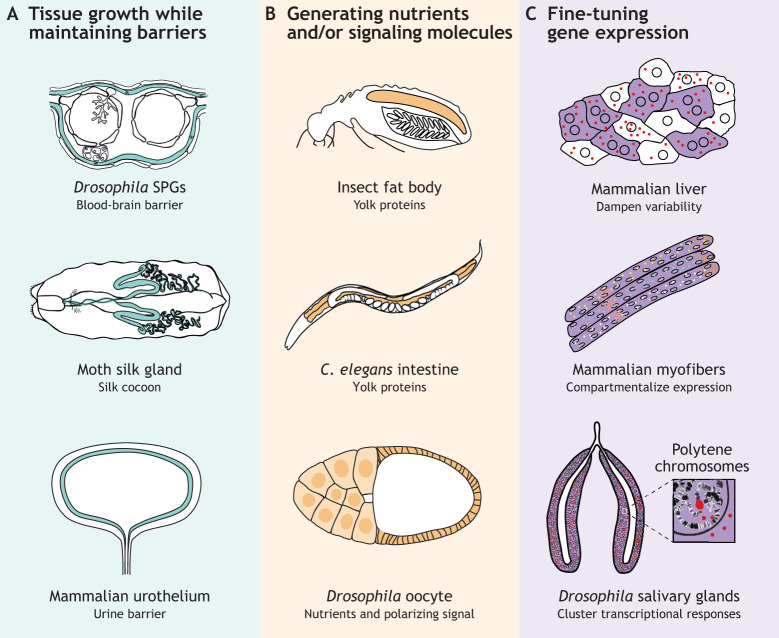
**Functions of naturally occurring (programmed) polyploid cells.** (A) Examples of polyploid cell types that need to maintain tissue barriers while growing. (B) Examples of polyploid tissues known to be important for the generation of nutrients and/or signaling molecules. (C) Known examples of how polyploid cells can fine tune tissue-specific gene expression. Colored spots in the cells represent mRNA transcripts. In the mammalian liver, polyploidy has been suggested to dampen variability in gene expression between cells (top image). In myofibers, multinucleation is used to compartmentalize gene expression, allowing different genes to be expressed in distinct locations in the muscle (middle image). In polytene nuclei, gene amplification allows transcriptional clustering, likely increasing transcriptional output of tissue-specific transcripts (bottom image).

There are also many organs and tissues that increase in size without becoming polyploid, by increasing cell proliferation and non-replicative growth (Amodeo and Skotheim, 2016). This raises the question of whether growth by polyploidy infers specific advantages for cells and tissues. One hypothesis is that polyploidy may be of particular advantage to facilitate the growth of cells and tissues that need to maintain a barrier because it allows for growth without disruption of cellular connections by cytokinesis. This function of polyploidy is nicely illustrated in the *Drosophila* blood-brain barrier, where SPGs are located underneath a dense extracellular matrix and perineurial sheath of cells and establish septate junctions with each other to form a functional barrier (Unhavaithaya and Orr-Weaver, 2012) (Fig. 2A). Leakage of fluorescently labeled dyes into the nervous system of flies is drastically increased in mutants of septate junction components, illustrating that these cellular connections are essential for the barrier function of the SPGs (Stork et al., 2008). Cell division would likely require the disassembly of septate junctions and disrupt the integrity of the blood-brain barrier. As SPGs need to grow during development to match the growth of the brain lobes, polyploidy provides an alternative mechanism to facilitate growth while maintaining intact tissue barriers (Unhavaithaya and Orr-Weaver, 2012; von Stetina et al., 2018). In fact, either an increase or decrease in SPG ploidy can disrupt septate junctions and compromise barrier function, suggesting an optimal ratio between cellular ploidy and organ size (Li et al., 2017; Unhavaithaya and Orr-Weaver, 2012). Consistently, SPGs that cover the larger brain lobes have a higher DNA content than those in the ventral cord, which cover a smaller surface area (Unhavaithaya and Orr-Weaver, 2012). Differences in cellular ploidies between different regions of an organ are likely also important to achieve specific organ shapes and functions, as has recently been reported in *Drosophila* and human hearts (Chakraborty et al., 2023).

There are many other polyploid tissues or cell types that form functional barriers, such as moth silk glands (Buntrock et al., 2012; Patrick Gage, 1974) (Fig. 2A), mammalian urothelial superficial cells (Wang et al., 2018) (Fig. 2A), mammalian keratinocytes (Gandarillas and Freije, 2014), *C. elegans* hypodermis and intestines (Hedgecock and White, 1985), insect larval abdominal epithelium (Bischoff and Cseresnyés, 2009; Nardi et al., 2018), insect Malpighian tubules (Buntrock et al., 2012; Rangel et al., 2015; Wang and Spradling, 2020), mammalian and insect salivary glands (Follette et al., 1998; Matsumoto et al., 2020), insect intestines (Fox et al., 2010; Schoenfelder et al., 2014; Scholes et al., 2014), mollusk albumen glands (Anisimov, 2005), mammalian pancreas (Webb et al., 1982) and mammary glands (Rios et al., 2016). In addition, many polyploid extra-embryonic tissues that play a role in providing nutrients to embryos are also important for creating a barrier or supporting the embryo micro-environment; for example, fish syncytial yolk nuclei (Kageyama, 1996; Kondakova and Efremov, 2014), mammalian trophoblast giant cells (Barlow and Sherman, 1974; Chen et al., 2012; Sher et al., 2013; Velicky et al., 2018; Zybina and Zybina, 2005, 2011, 2020) or syncytiotrophoblasts (Azar et al., 2018) and *Drosophila* follicle cells (Maines et al., 2004). Interestingly, many polyploid cell types undergo polyploidization during either postnatal development or reproduction, two periods in the life cycle when tissues need to grow and function simultaneously. Thus, polyploidization likely facilitates the growth of tissues without disrupting important functions that would be compromised by cell division, such as the formation of a barrier and maintenance of a microenvironment. Additional experiments will be needed to provide further support of this hypothesis, ideally in an experimental system where it is possible to perform tissue-specific cell-cycle manipulations and compare tissues formed by polyploid cells versus tissues formed by a multitude of diploid cells. This would provide definitive proof of whether the generation of a tissue solely by canonical cell divisions and thus diploid cells would result in compromised tissue barriers or organ malfunction.

## Polyploid cells generate nutrients and signaling molecules for their surroundings

### Polyploid cells supply nutrients

In many different plant and animal species, polyploid cells generate macronutrients that are required to support the growth of the offspring. A well-studied example is the insect fat body (Arrese and Soulages, 2010), an insect-specific tissue that is distributed throughout the body cavity and consists mainly of adipocytes in which energy reserves are stored in the form of glycogen and triglycerides (Fig. 2B). The fat body plays multiple roles in insect physiology by facilitating energy storage and utilization, but also by producing proteins and metabolites that enter circulation. The fat body is not only responsible for providing energy during extended periods of starvation but is also essential for the generation and loading of lipids into developing oocytes. During oogenesis, the fat body produces large amounts of yolk proteins, also known as vitellogenins, which are a specific type of lipid transport protein expressed in females of nearly all egg-laying species. Vitellogenins mobilize lipids from the fat body and transport them to the developing oocytes (Fig. 2B). Fat body polyploidization has been characterized in many different species, such as flies (*D. melanogaster*; Juhász and Sass, 2005), mosquitos (*Aedes aegypti*; Dittmann et al., 1989), ants (*Dinoponera australis*; Scholes et al., 2014), termites (six different species; Nozaki and Matsuura, 2016, 2019), moths (*Bombyx mori*; Chinzei and Tojo, 1972) and locusts (*Locusta migratoria*; Irvine and Brasch, 1981; Nair et al., 1981). The functional importance of polyploidy in the fat body is nicely illustrated by studies in termites, where there is a strong positive correlation between fat body DNA content and fecundity in termite queens (Nozaki and Matsuura, 2016, 2019). Similarly, polyploidization of the locust fat body is also important for reproductive fitness because blocking polyploidization results in reduced vitellogenin expression, impaired oocyte maturation and arrested ovarian development (Wu et al., 2020).

In addition to fat bodies, there are other polyploid tissues known to provide nutrients for the offspring: *C. elegans* intestinal cells produce vitellogenins that are secreted and taken up by developing oocytes (Kimble and Sharrock, 1983) (Fig. 2B); *Drosophila* nurse cells synthesize nutrients and cytoplasmic components that are transported into the oocyte (Bastock and St Johnston, 2008) (Fig. 2B); plant endosperm and pericarp support the growth of the seed embryo (Boisnard-Lorig et al., 2001; Bourdon et al., 2012; Chevalier et al., 2011; Sabelli and Larkins, 2009); mammalian trophoblast giant cells facilitate early transport and exchange of nutrients (Chen et al., 2012; Zybina and Zybina, 2020); and mammary glands produce milk for the offspring (Rios et al., 2016). Whether polyploidy is essential for nutrient production in these tissues remains to be determined. Preventing cell-cycle progression in the trophoblast or mammary glands gives profound growth defects in the progeny, suggesting that polyploidization is important for nutrient provision to the offspring (Geng et al., 2003; Rios et al., 2016). However, polyploidization per se might not be essential for nutrient production, because replacing endoreplication cycles with canonical cycles in mouse trophoblast does not give rise to embryonic defects (Chen et al., 2012). Thus, polyploidization may facilitate the large-scale production of nutrients, but this function can likely also be fulfilled by the generation of additional diploid cells.

### Polyploid cells generate signaling molecules

Polyploid cells can also alter or support their surroundings by secreting signaling molecules that control patterning and stem cell homeostasis. The polyploid *Drosophila* follicle cells, which form a layer around the developing oocyte and the nutrient-providing nurse cells, produce a yet unidentified signal that defines the anteroposterior axis of the embryo (Doerflinger et al., 2022; Gonzâlez-Reyes et al., 1995; Roth et al., 1995) (Fig. 2B). Similarly, polyploid nuclei of the fish yolk syncytial layer are a source of inductive signals, such as Nodal/TGFβ ligands, which are required for the formation of the three germ layers (Carvalho and Heisenberg, 2010). Polyploid glial cells in the *Drosophila* blood-brain barrier, next to serving a barrier function, also control neural stem cell proliferation by secreting insulin, alanine and lactate in response to nutritional cues (Chell and Brand, 2010; Spéder and Brand, 2014). The secretion of nutrients and metabolites by polyploid glia is required for neuronal stem cells to exit quiescence in response nutritional cues, and also more generally to maintain healthy neurons, as a lack of glial glycolysis causes neurodegeneration (Chell and Brand, 2010; Spéder and Brand, 2014). Similarly, the polyploid *C. elegans* hypodermis is required to reactivate quiescent blast cells upon nutrient stimulation (Fukuyama et al., 2015).

In mammals, trophoblast giant cells, hepatocytes and pancreatic cells all have distinct signaling functions for which polyploidization could be important: trophoblast giant cells secrete a wide range of hormones and cytokines that instruct maternal adaptations during pregnancy (Hu and Cross, 2010), hepatocytes play an important role in innate immunity by secreting immune-modulating factors (Zhou et al., 2016) and the primary function of pancreatic B cells is the production and secretion of hormones that regulate blood glucose levels, such as insulin and amylin (Marchetti et al., 2017). Surprisingly, tissue-specific inhibition of polyploidization in these tissues does not result in gross physiological defects but only impairs tissue functioning under stress conditions (Chen et al., 2012; Matondo et al., 2018; Pandit et al., 2012). Thus, an increase in ploidy could be particularly important for tissue function under suboptimal conditions, which may explain the higher numbers of polyploid cells in the pancreas of diabetic mice and in livers of animals after partial hepatectomy, oxidative damage or metabolic and toxic stresses (Pohl and Swartz, 1979; Wang et al., 2017).

## The role of polyploidy in gene-expression control

Recent advances in single-cell transcriptomics are beginning to shed light on the effect of polyploidy on gene expression. Differences in gene expression have been reported between diploid and polyploid cells in the mammalian liver (Lu et al., 2007; Richter et al., 2021), blood (Choudry et al., 2021; Sun et al., 2021) and heart (Windmueller et al., 2020; Yekelchyk et al., 2019). For example, polyploid cardiomyocytes have relatively lower expression of cell-cycle genes and higher expression of genes involved in cardiomyocyte maturation, such as sarcomere organization and fatty acid oxidative metabolism (Windmueller et al., 2020). Furthermore, studies in the mammalian liver suggest that polyploidy may function to dampen the intrinsic variability associated with gene transcription, as polyploid hepatocytes exhibit less gene expression noise than diploid hepatocytes (Bahar Halpern et al., 2015; Richter et al., 2021) (Fig. 2C). It is currently unclear whether the transcriptional differences observed between diploid and polyploid cells are a direct consequence of polyploidization or arise in response to other cues associated with ageing and/or maturation, because polyploidy increases during maturation of hepatocytes, megakaryocytes and cardiomyocytes (Pandit et al., 2013). Nonetheless, it is not unlikely that polyploidy influences gene expression because having multiple copies of the genome inherently alters the way DNA is organized in the cell. For example, having multiple genomes within one nucleus likely alters the nuclear surface-to-volume ratio and which fraction of the genome is in contact with the nuclear lamina.

### Multinucleation fine-tunes tissue-specific gene expression

Evidence for a function of polyploidy to compartmentalize gene expression in tissues comes from recent studies in mouse skeletal muscle in which differentiated mononucleated muscle progenitor cells fuse to form myofibers: very large polyploid syncytia that contain hundreds of nuclei in a shared cytoplasm (Abmayr and Pavlath, 2012). Single-nucleus RNA sequencing and RNA fluorescence *in situ* hybridization (FISH) have revealed distinct gene expression profiles between individual nuclei of myofibers (dos Santos et al., 2020; Kim et al., 2020; Petrany et al., 2020). Even so, nuclei within one myofiber can coordinate their transcriptional activity to simultaneously express specific myosin heavy chain isoforms (dos Santos et al., 2020; Kim et al., 2020). Strikingly, nuclei from different myofibers in specific regions of the muscle can also coordinate their gene expression, a process that is regulated by muscle innervation (dos Santos et al., 2020). Together, these findings demonstrate that multinucleation can be used to compartmentalize gene expression in large, syncytial cells (Fig. 2C).

In addition to allowing spatial control of gene expression, multinucleation may also be beneficial for other aspects of transcription (Peterson and Fox, 2021). A comparison of binucleated and mononucleated *C. elegans* intestinal cells suggests that binucleation is important for rapid transcriptional responses (van Rijnberk et al., 2022). In animals with mononucleated instead of binucleated intestinal cells, gene expression is less efficient, despite cells having the same total DNA content (either one 64C nucleus or two 32C nuclei) (van Rijnberk et al., 2022). It is possible that in highly polyploid nuclei, transcription becomes limiting due to restricted nuclear transport of transcriptional regulators, making multinucleation advantageous to boost transcriptional responses. An alternative mechanism to maximize transcription in polyploid cells could be to increase the nuclear surface-to-volume ratio by changing the shape of polyploid nuclei. Indeed, some polyploid nuclei, such as those of silk glands, megakaryocytes, trophoblast giant cells and tomato fruit cells, have highly lobulated nuclei (Bourdon et al., 2012; Buntrock et al., 2012; Henderson and Locke, 1991; Klisch et al., 2005; Nagata et al., 1997). Whether and how changes in nuclear shape influence transcription in polyploid cells remains to be determined.

### Clustering of amplified genes in polytene nuclei

Polyteny likely also confers transcriptional advantages. In polytene polyploid nuclei, DNA loci remain closely associated, often creating characteristic banding patterns with silenced heterochromatic regions visible by light microscopy as darkly stained bands, which are interspersed with lighter-stained euchromatic regions (Stormo and Fox, 2017). Polytene *Drosophila* salivary glands are widely used as a model system to study gene expression and chromatin organization, and it has long been appreciated that changes in gene expression, for example due to hormone pulses or heat-shock, can visibly alter polytene chromosome structures, creating ‘puffs’ in the polytene chromosomes in which the transcription machinery accumulates (Andrulis et al., 2000; Breuer and Pavan, 1955; Ritossa, 1962; Stormo and Fox, 2017; Yao et al., 2006, 2007) (Fig. 2C). Thus, the amplification of specific gene loci, such as chorion and vitelline genes in *Drosophila* follicle cells (Kim et al., 2011; Spradling, 1981) or genes involved in mammalian placenta development in trophoblast giant cells (Hannibal and Baker, 2016), likely allows polyploid nuclei to cluster many gene copies, thereby enhancing tissue-specific gene expression (Fig. 2C). Nonetheless, there is no experimental evidence showing that the organization into polytene chromosomes is essential for polyploid tissue function. It is also possible that polyteny arises as a strategy to shorten S-phase by not replicating the entire chromosome, and that it is not particularly advantageous for tissue-specific gene expression. This is supported by the notion that under-replicated regions are the same between different *Drosophila* polytene tissues, such as fat bodies, ovaries, midguts and salivary glands, and that there is no obvious phenotype in mutants with altered polytene organizations (Belyaeva et al., 1998; Nordman et al., 2011; Yarosh and Spradling, 2014). Thus, it remains to be determined what the specific advantages are of polyteny.

### Future avenues to investigate gene expression in polyploidy

Having multiple copies of the genome will inevitably alter gene expression in polyploid cells. Deciphering these changes however is not trivial, as most sequencing-based technologies measure relative and not absolute changes in gene expression. Moreover, to understand how specific polyploid spatial genome organizations influence gene expression, one would need to compare cells that have the same ploidy but different genome organizations. These types of experiments are starting to become feasible by advances in targeted protein disruption that allow cell-cycle regulators to be depleted in specific tissues during limited moments in development (Campbell and Bennett, 2016; van Rijnberk et al., 2022). Combining these manipulations with single-cell and single-molecule imaging approaches to measure gene expression will elucidate how genome amplification affects gene expression and provide insights whether specialized genome structures in polyploid cells are important for fine-tuning gene expression.

## Protective roles of polyploid cells in tissue homeostasis

### Polyploid cells are less sensitive to DNA damage

Somatic polyploidy not only appears to be important to increase tissue growth and enhance protein production, it also likely has protective functions. Intuitively, one could argue that having multiple genome copies within one cell likely protects polyploid cells against disruptive DNA mutations, although this has not been experimentally proven. Nonetheless, naturally occurring polyploid cells are more resistant against DNA damage (Davoli and De Lange, 2011; De Renty et al., 2014; Hassel et al., 2014; Mehrotra et al., 2008; Nandakumar et al., 2020; Zhang et al., 2014). This is likely due to a global repression of the machinery that induces apoptosis in response to DNA damage in polyploid cells, such as p53 and the checkpoint protein kinase CHK1 (Davoli and De Lange, 2011; Mehrotra et al., 2008; Ullah et al., 2008; Zhang et al., 2014). How polyploid cells repress the genes involved in apoptosis signaling is currently unclear, but the effect is independent of cellular differentiation (Hassel et al., 2014). It is possible that polyploid cells have evolved mechanisms to repress the normal apoptotic response to unrepaired DNA damage to avoid double-strand DNA breaks by stalled replication forks at under-replicated regions (Mehrotra et al., 2008). More research is needed to elucidate the mechanism and function of the altered DNA damage response of polyploid cells.

### Polyploidy protects against overproliferation

Most naturally occurring polyploid cells lose their capacity to proliferate. It is unclear whether polyploidy itself induces cell-cycle exit or whether their stop in proliferation occurs independently as part of a terminal differentiation program. Several polyploid cell types have been shown to lose their centrioles during polyploidization, making it difficult for these cells to undergo subsequent mitotic divisions (Lu and Roy, 2014; Mahowald and Strassheim, 1970; Mahowald et al., 1979; Schoenfelder et al., 2014; Tassin et al., 1985). Due to the strong correlation between becoming polyploid and losing proliferative capacity, it is possible that polyploidy has an important function in tissues to prevent over-proliferation. This appears to be the case in the liver, which is exposed to a large number of toxins and where hepatocyte polyploidy protects against tumorigenesis (Lin et al., 2020; Wilkinson et al., 2019; Zhang et al., 2018). Interestingly, hepatocytes are one of the few polyploid cell types that can continue to proliferate after becoming polyploid (Duncan et al., 2010). Nonetheless, the vast majority of human hepatocellular carcinoma (HCC) are diploid, further supporting a cancer-preventive effect of polyploid cells. This cancer-protective function of polyploid hepatocytes has been attributed to the reduced chances of losing tumor suppressor genes in polyploid cells, as well as to reduced proliferative capacity of polyploid cells (Lin et al., 2020; Wilkinson et al., 2019; Zhang et al., 2018).

## Consequences of stress-induced polyploidization

Polyploidization not only occurs as part of normal development in specialized cell types and tissues, but it can also arise under pathological conditions in cells that would normally remain diploid. For example, polyploidy arises in response to injury, infection (Box 1) or DNA-damaging agents or cell division failures. In the mid-1800s, giant polyploid cells were identified in tumor tissues, as well as in tuberculosis and syphilis lesions (Editorial, 1877; Editorial, 1878; McCarthy, 1980). Despite their established presence in many different diseases, the contribution of polyploid cells in disease progression has only recently begun to be addressed.

**Box 1. Polyploidy and parasitic infection**

**Fig. 3.**
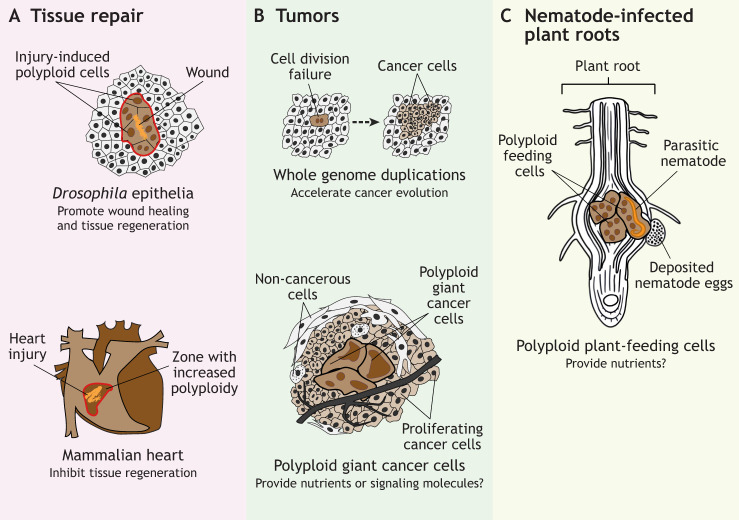
**Examples of polyploid cells that arise in response to ectopic stresses and their hypothesized functions.** (A) In wounded tissues, injuries (depicted in orange) can result in regions with increased polyploidy (outlined in red), which can either be beneficial (top) or detrimental (bottom) for tissue regeneration. (B) In tumorigenesis, cell division failure can give rise to polyploid cells (depicted in brown) that either evolve into highly proliferative cells (top) or become polyploid giant cancer cells (bottom), that potentially provide nutrients and signaling molecules to surrounding cancer cells (light brown cells). (C) In plant roots that are infected by parasitic nematodes (depicted in orange), plant cells become polyploid (brown cells) and function as feeding sites for nematode growth. Diploid cells or tissues are depicted in white/light grey.

A supporting role of polyploid cells in pathogenesis is found in infiltration of plants by parasitic nematodes, which induce polyploidization of plant root cells (De Almeida Engler and Gheysen, 2013; Gheysen and Fenoll, 2003). Root-knot and cyst nematodes induce endoreduplication or cell fusion in plant roots to form multinucleated polyploid cells that develop into nematode-feeding sites (Fig. 3C). These feeding cells undergo major developmental reprogramming, including differential expression of genes related to metabolism, stress response, protein synthesis, transport and signal transduction, creating a nutrient-rich and non-hostile environment for nematode growth (Gheysen and Fenoll, 2003). Moreover, neighboring plant cells undergo increased proliferation, leading to the formation of the typical root gall (De Almeida Engler et al., 1999; Golinowski et al., 1996). Interestingly, the ability to induce polyploidization in plant root cells, either by cell fusion or by non-canonical cell cycles, evolved multiple times in different clades of nematodes (Smant et al., 2018), strongly suggesting that the generation of polyploid cells in the plant root provides a fitness advantage for nematodes. More research is needed to understand why polyploidization, and not overproliferation of root cells, for example, is advantageous for parasitic nematode growth. It is possible, for example, that the large polypoid plant cells are better at generating large quantities of nutrients. Alternatively, the large polyploid cells could provide a large and protected environment in which nematodes can grow, without completely disrupting the root tissue.

### Effects of injury-induced polyploidy in tissue repair

Polyploidization can occur in response to tissue injury or cellular stress, when cells are lost and cell mass needs to be restored. In many organs, injury activates tissue-resident stem cells to replace lost cells by cell division (Blanpain and Fuchs, 2014). However, in some organs and tissues, particularly tissues that lack progenitor or stem cells, post-mitotic cells become polyploid during the wound healing process. For example, in multiple *Drosophila* epithelia, injury induces polyploidization of cells near the wound site, which is important for wound healing (Cohen et al., 2018; Galko and Krasnow, 2004; Losick et al., 2013; Xiang et al., 2017) (Fig. 3A). This induced polyploidization can be either by cell fusion or by endoreplication cycles, both of which stimulate hypertrophic growth. The importance of polyploidy for tissue regeneration is not completely understood and will require additional research in experimentally tractable model systems such as the *Drosophila* hindgut pylorus, in which ploidies can be altered and cellular responses can be monitored during tissue repair. Here, functional analyses revealed that an increase in either diploid or polyploid cells can repair tissues after injury but, in response to sustained growth signaling, only polyploid cells preserve epithelial architecture (Cohen et al., 2018).

Tissue injury also induces polyploidization in vertebrate hearts, livers, kidneys, and lungs (De Chiara et al., 2022; Donne et al., 2020; Ebert and Pfitzer, 1977; Lazzeri et al., 2018; Senyo et al., 2012; Weng et al., 2022). In these different tissues, the effects of polyploidization after injury can vary substantially. For example, in injured mouse and human hearts, polyploid cardiomyocytes appear to impair regeneration (González-Rosa et al., 2018; Patterson et al., 2017). Conversely, in the injured zebrafish heart, polyploidization of a subset of epicardial cells that surround the heart is thought to promote wound healing and repair (Cao et al., 2017). In the liver, injury-induced polyploidy neither inhibits nor promotes regeneration (Pandit et al., 2012). It is currently unclear what determines the effect of polyploid cells during tissue repair and why, in some cases, polyploidy can be beneficial, whereas in others it inhibits regeneration. Many parameters such as differentiation status, epithelial organization and cell density will likely influence the wound healing and repair process, making it technically challenging to pinpoint the specific contribution of polyploidy.

### Polyploidy by cell division failure leads to decreased cellular fitness

In general, cells that fail to divide and become polyploid are usually less fit. This has been mostly characterized in animal cells, where induction of cytokinesis failure and concomitant tetraploidization leads to cell-cycle arrest or apoptosis, a process dependent on p53 signaling (Gerlach et al., 2018; Storchova and Kuffer, 2008); but also in single-cell organisms such as budding yeast, which lack p53, where polyploidization leads to slow growth and genetic instability (Andalis et al., 2004; Mable, 2001; Mayer and Aguilera, 1990; Mozzachiodi et al., 2022; Storchová et al., 2006). These defects are thought to arise from imbalances in protein content in polyploid cells: despite cell volume linearly increasing with cell ploidy, protein content shows sublinear scaling, and protein translation is reduced in polyploid yeasts (Yahya et al., 2022). By contrast, polyploidization of human cells grown in culture does not give rise to gross differences in protein concentrations (Lanz et al., 2022). Nonetheless, immediately after becoming polyploid, cells have trouble accumulating enough proteins to correctly replicate their DNA, giving rise to under- and over-replicated regions and genetic instability (Gemble et al., 2022). Taken together, cells that become polyploid after cell division failure become less fit due to protein content imbalances and slow growth. Paradoxically, a polyploidization event (in this context often referred to as WGD) is thought to be an early and important step during tumor evolution, likely by facilitating genetic diversification and promoting cancer development (summarized in Box 2). How cell division failure can provide a fitness advantage to a subset of cells and give rise to tumors is an active field of current investigation (Box 2).

**Box 2. Effects of whole genome doubling on cell proliferation and tumorigenesis**

The first compelling proof that polyploidy promotes cellular transformation and tumor formation came from mouse experiments in which either diploid or tetraploid *p53^–/–^* cells were transplanted into immunocompromised mice, and only the tetraploid cells gave rise to malignant cancers (Fujiwara et al., 2005). Since then, sequencing analyses of thousands of cancer genomes have confirmed an important contribution of polyploid cells in tumors, and the occurrence of polyploidy is associated with a worse prognosis (Bielski et al., 2018; Priestley et al., 2019; Zack et al., 2013). After polyploidization, cells are more prone to accumulate DNA damage and become genomically unstable (Lens and Medema, 2018; Storchova and Pellman, 2004). There is accumulating evidence that this polyploidy-induced chromosomal instability underlies the formation of aneuploid cells, which have abnormal chromosome contents (either gains or losses of whole chromosomes or chromosome fragments) and promote tumorigenesis (Fujiwara et al., 2005; Galipeau et al., 1996; López et al., 2020; Prasad et al., 2022; Watkins et al., 2020) (Fig. 3B). Furthermore, recent work suggests that WGD in cancer cells drives chromatin organization changes, thereby changing transcriptional activities that likely promote oncogenic transformation (Lambuta et al., 2023).

Many cancers contain polyploid giant cancer cells (PGCCs) and their role in tumor aggressiveness and relapse after therapy has recently been gaining attention (Herbein and Nehme, 2020; Pienta et al., 2022; Sikora et al., 2020; Tagal and Roth, 2021; Zhang et al., 2021). It has been suggested that PGCCs could play a role in creating a microenvironment for tumor development by generating nutrients and growth factors (Fajka-Boja et al., 2018, 2020; Schweizer et al., 2015) (Fig. 3B). Thus, similar to a role of polyploid cells in development, polyploid cells could also influence surrounding cells and tissues during tumorigenesis.

## Conclusions and future directions

Analyses of somatic polyploidy in a plethora of different multicellular organisms have made it clear that polyploidization is common in tissues that form epithelial barriers, as well as in cells that are highly transcriptionally and metabolically active. Despite the long-observed notion that polyploid cells are large and tend to generate nutrients or signaling molecules for their surroundings, empirical studies addressing how polyploidization affects cell growth rates, mRNA transcription, protein synthesis and nutrient secretion are largely lacking. Future research in experimentally tractable systems, such as polyploid tissues of *Drosophila* and *C. elegans*, will likely deepen our understanding of the consequences of polyploidy in tissue homeostasis and disease. In these systems, ploidies can be manipulated in specific cell types during defined moments of development, and phenotypes can be characterized in great detail by microscopy and single-cell sequencing approaches. This will allow researchers to answer open questions such as: how do RNA transcription, protein synthesis and cell growth scale with polyploidy? Do polyploid cells have altered protein secretion and/or uptake? Are the molecular and cellular consequences of polyploidy different between cells that become polyploid during normal development versus cells that become polyploid by ectopic stresses? Defining the roles of polyploid cells in different contexts will increase our knowledge of how specialized tissues function in homeostasis and disease, ultimately providing insights to predict disease outcomes and responses to therapies.
